# Enhanced paracellular transport of insulin can be achieved via transient induction of myosin light chain phosphorylation

**DOI:** 10.1016/j.jconrel.2015.05.270

**Published:** 2015-07-28

**Authors:** Alistair Taverner, Ruggero Dondi, Khaled Almansour, Floriane Laurent, Siân-Eleri Owens, Ian M. Eggleston, Nikoletta Fotaki, Randall J. Mrsny

**Affiliations:** a Department of Pharmacy and Pharmacology, University of Bath, Bath BA2 7AY, UK; b Welsh School of Pharmacy, Cardiff University, Cardiff, CF10 3XF, UK

**Keywords:** CPP, Cell Penetrating Peptide, DAPI, 4′,6-diamidino-2-phenylindole, FD, Fluorescent dextran, FMC, 9-fluorenylmethyloxycarbonyl, ILI, Intraluminal injection, MLC, Myosin light chain, MTS, 3-(4,5-dimethylthiazol-2-yl)-5-(3-carboxymethoxyphenyl)-2-(4-sulfophenyl)-2H-tetrazolium, pMLC, Phosphorylated myosin light chain, PBS, Phosphate buffer saline, PK/PD, Pharmacokinetics/pharmacodynamics, MLCK, Myosin light chain kinase, MLCP, Myosin light chain phosphatase, MYPT1, Myosin phosphatase target subunit, PKC, Protein kinase C, SC, Subcutaneous, SPPS, Solid phase peptide synthesis, TJ, Tight junction, Paracellular transport, Myosin light chain phosphatase, Insulin delivery, Cell penetrating peptide, Protein–protein interactions, Tight junction function

## Abstract

The intestinal epithelium functions to effectively restrict the causal uptake of luminal contents but has been demonstrated to transiently increase paracellular permeability properties to provide an additional entry route for dietary macromolecules. We have examined a method to emulate this endogenous mechanism as a means of enhancing the oral uptake of insulin. Two sets of stable Permeant Inhibitor of Phosphatase (PIP) peptides were rationally designed to stimulate phosphorylation of intracellular epithelial myosin light chain (MLC) and screened using Caco-2 monolayers in vitro. Apical application of PIP peptide 640, designed to disrupt protein–protein interactions between protein phosphatase 1 (PP1) and its regulator CPI-17, resulted in a reversible and non-toxic transient reduction in Caco-2 monolayer trans-epithelial electric resistance (TEER) and opening of the paracellular route to 4 kDa fluorescent dextran but not 70 kDa dextran in vitro. Apical application of PIP peptide 250, designed to impede MYPT1-mediated regulation of PP1, also decreased TEER in a reversible and non-toxic manner but transiently opened the paracellular route to both 4 and 70 kDa fluorescent dextrans. Direct injection of PIP peptides 640 or 250 with human insulin into the lumen of rat jejunum caused a decrease in blood glucose levels that was PIP peptide and insulin dose-dependent and correlated with increased pMLC levels. Systemic levels of insulin suggested approximately 3–4% of the dose injected into the intestinal lumen was absorbed, relative to a subcutaneous injection. Measurement of insulin levels in the portal vein showed a time window of absorption that was consistent with systemic concentration-time profiles and approximately 50% first-pass clearance by the liver. Monitoring the uptake of a fluorescent form of insulin suggested its uptake occurred via the paracellular route. Together, these studies add validation to the presence of an endogenous mechanism used by the intestinal epithelium to dynamically regulate its paracellular permeability properties and better define the potential to enhance the oral delivery of biopharmaceuticals via a transient regulation of an endogenous mechanism controlling the intestinal paracellular barrier.

## Graphical abstract

**Figure:**
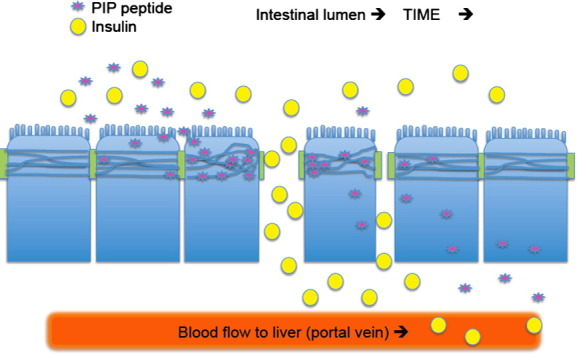

## 1 Introduction

Oral peptide delivery has been a goal for the pharmaceutical industry for decades; soon after the identification of insulin as a treatment for diabetes, efforts were made to attempt its therapeutic administration following oral delivery [1]. A physiological rationale exists for this strategy in the case of certain biopharmaceuticals, such as insulin, as oral uptake would result in direct delivery to the liver via the portal circulation, with the liver being the primary site of glucose regulation in the body [2]. Paramount to successfully achieving this goal is the sufficient stabilization of labile biopharmaceuticals following oral administration as they encounter the harsh environment of the stomach and enzymatic milieu of the small intestine. Protection during gastric transit can be achieved by enteric coating of the dosage form and agents generally regarded as safe [3] can be used to suppress peptidase activities in the small intestine [4]. Despite these efforts, only extremely low amounts of insulin are observed to transport across intestinal epithelia [5]. Thus, it is not surprising that a plethora of efforts have been described to enhance the transport rate of a biopharmaceutical by disrupting or disorganizing the tight junction (TJ) structures that restrict the flux of macromolecules between adjacent epithelial cells [6,7].

Polarized intestinal epithelial cells can dynamically ‘open’ and ‘close’ TJ structures through the reversible phosphorylation of a 20 kDa regulatory myosin light chain (MLC) protein; the set position for MLC is de-phosphorylated to keep TJs in a ‘closed’ state [8]. Closed TJs limit the paracellular uptake of hydrophilic agents with a size greater than < 15 Å, which equates to a molecular mass of ~ 3.5 kDa [9]. Transient TJ “opening” to enhance paracellular nutrient uptake, however, has been suggested as a natural phenomenon of intestinal physiology [10] and increased levels of phosphorylated MLC are associated with open TJs [11]. Since MLC phosphorylation is dynamically regulated in polarized epithelial cells by a specific kinase (MLCK) and a specific phosphatase (MLCP), we have examined methods to selectively block MLCP function as a means to transiently increase MLC phosphorylation by basal MLCK activity. The role of MLC phosphorylation in regulating TJ paracellular permeability properties was previously validated using a d-amino acid, membrane-permeable peptide, termed PIK that selectively inhibits active MLCK activity [12–14].

MLCP is a trimeric complex consisting of a protein phosphatase-1 (PP1) isoform, the myosin targeting subunit MYPT1-CPI-17 regulatory complex, and a 21 kDa accessory subunit [15–17]. We examined the potential for rationally designed small peptides that emulate specific MLCP holoenzyme domains involved in protein–protein interfacial contacts to regulate its catalytic activity and thereby affect TJ-mediated barrier function. To identify potential Permeant Inhibitor of Phosphatase (PIP) peptides, we focused on interactions between MYPT1 or CPI-17 with PP1. We assumed that differences in MLCP regulation by Rho kinase versus protein kinase C (PKC) pathway activation would be recapitulated by PIP peptides that disrupted interactions between PP1 and MYPT1 or CPI-17, respectively. Thus, the effectiveness and duration of action of such PIP peptides should be dependent upon several factors: their biochemical stability, access to cytoplasmic MLCP in intestinal epithelial cells, residence time at specific protein–protein interfacial surfaces, and the role of that protein–protein interaction in MLCP function. We now report the identification two rationally designed PIP peptides that are capable of dynamically opening TJs in vitro and that enhance the uptake of biologically active insulin in vivo. These results further refine our understanding of how specific protein–protein interactions within the MLCP holoenzyme may regulate its functional properties in intestinal epithelial cells.

## 2 Materials and methods

### 2.1 Peptide synthesis

Peptides were synthesized by (Fmoc)-SPPS using amino acid derivatives obtained from Novabiochem, except for isoleucine, which was obtained from Sigma Aldrich. The first amino acid was coupled to Rink Amide MBHA resin (100–200 mesh; Novabiochem) using *N*,*N*′-diisopropylcarbodiimide and 1-hydroxbenzotriazole [18]. Subsequent couplings were carried out on an Activo P-11 peptide synthesizer using PyBOP. Deprotection was carried out using 20% piperidine in dimethylformamide. Peptides were cleaved from the resin using trifluoroacetic acid (TFA), triisopropylsilane and water (95:2.5:2.5) [19], and precipitated in diethyl ether. Crude product was purified by HPLC, using a Phenomenex Gemini C18 column (250 × 10 mm, pore size 5 μm) and a gradient mobile phase of water and acetonitrile (both with 0.1% TFA) using a flow rate of 2.5 mL/min. High-resolution time-of-flight mass spectra were obtained on a Bruker Daltonics micrOTOF mass spectrometer using electrospray ionization (ESI) to verify peptide identity. Purified peptides were lyophilized and stored at − 20 °C.

### 2.2 Cell culture

An immortalized human intestinal epithelial cell line (Caco-2) was maintained in DMEM/F12 (Gibco, Paisley, UK) supplemented with 10% FBS, 2 mM l-glutamine (Gibco) 100U/mL penicillin and 100 μg/mL streptomycin (Gibco). Caco-2 cells were seeded at a density of 7 × 10^4^/well on Transwell™ (Corning, NY) polyester membrane filters (12 mm diameter, 0.4 μm pore size). Feeding with fresh media (Life Technologies, Paisley, UK) was carried out every second day [20]. Caco-2 monolayers with trans-epithelial electrical resistance (TEER) > 350 Ω·cm^2^, as measured using fixed paddle electrodes and a voltohmeter (World Precision Instruments©, UK) that was typically achieved between days 15–18 following seeding on Transwell™ filters were used for these studies.

### 2.3 In vitro transport studies

Apical to basal flux of 50 mg/ml 4 kDa dextran or 50 mg/mL 70 kDa dextran (Sigma) was performed to assess the impact of PIP peptides on paracellular permeability [21]. Apical (200 μL) and basal (600 μL) compartment media were replaced with HBSS and allowed to equilibrate for 30 min; TEER measurements were obtained prior to use to ensure monolayer integrity [22]. After apical application of the dextrans the basal compartment volume was collected at set times (typically 0, 15, 30 60, 90, and 180 min) and replaced with fresh HBSS. Apical and basal compartment fluorescence was determined using a Fluorostar Omega microplate reader (BMG Labtech, Ortenburg, Germany). After 3 h, the apical compartment dextran and peptide solution was removed and replaced with PBS and TEER values were recorded for a further 30 min to assess monolayer recovery. TEER values were calculated by subtracting blank filter readings and normalized as a percentage of the initial TEER value for that monolayer [20].

### 2.4 In vivo studies

Male Wistar rats were housed in groups of 3–5 per cage with a 12/12 h light/dark cycle and weighed 225–275 g (approximately 6–8 weeks old) when placed on study. All experiments were conducted during the light phase with animals having ad lib access to food and were carried out using a non-recovery protocol that used continuous isoflurane anesthesia. Inhaled isoflurane was used instead of other forms of anesthesia that can affect blood glucose levels [23,24]. A 4–5 cm midline abdominal incision was made to expose the small intestine (mid-jejunum to proximal ileum regions) and to provide access to the portal vein for blood collection. Stock solutions of insulin (human recombinant; Sigma) and PIP peptides were prepared in phosphate buffered saline (PBS) containing 10 mM citric acid (pH 4.5) to reduce local proteolysis [4] and mixed 1:1 before injection using a 29-gauge hypodermic needle in a volume of 200 μL/kg (or ~ 50 μL per 250 g rat). The injection site mesentery was marked with a permanent marker. Blood draws were taken from the portal vein as well as systemic circulation over the next 2 h to measure glucose using a glucometer (AccuChek), serum insulin by ELISA (EMD Millipore Corp.), and endotoxin by Limulus Amebocyte Lystae (LAL) chromogenic assay (Thermo Scientific). Control treatment groups included intestinal injection of PIP peptide without insulin or insulin alone. At study termination, a 3–5 mm region that captured the marked intestine segment was isolated. This tissue was lysed for biochemical assessment or fixed, sectioned, and stained with hematoxylin/eosin prior to analysis read by KWS BioTest, a licensed veterinary pathologist. Subcutaneous (SC) insulin injections (20 μL/kg) were performed in the mid-scapular region with blood glucose and venous insulin levels being as above. All experiments were performed in accordance with the U.K. Animals (Scientific Procedures) Act of 1986, the European Communities Council Directive of 1986 (86/609/EEC), and the University of Bath's ethical review procedures.

### 2.5 PhosphoMLC analysis

After rinsing with ice cold PBS to remove PIP peptide or control treatment agents, isolated intestinal tissue was placed in ice cold PBS for 15 min prior to addition of 25 μL protease inhibitor cocktail (Fisher), 25 μL phosphatase inhibitor cocktail (Fisher) and 500 μL RIPA buffer (Sigma Aldrich). After 10 min on ice, lysates were centrifuged at 8000 rpm for 15 min to collect the supernatant, which was stored at − 80 °C until use. For Western blot analysis, lysates were separated by SDS-PAGE (12%) run at 220 V for 40 min and electro-transferred onto a PDVF membrane at 30 V for 70 min using an XCell™ Blot module (Invitrogen). Membranes were blocked using 5% bovine serum albumin in TBS-T (2 M Tris HCl, pH 7.5, 4 M NaCl and 0.1% Tween 20) for 1 h. Membranes were washed with water, incubated with primary antibody (anti-myosin light chain (phospho S19) antibody (Cell Signaling Technologies)) or anti-myosin light chain 2 antibody (Abcam) overnight at 5 °C, washed thrice with TBS-T, and then incubated with secondary horseradish peroxide (HRP)-coupled antibody for 1 h at room temperature. After washing thrice in TBS-T, HRP activity was detected by ECL (Santa Cruz).

### 2.6 Cell viability measurement

Induction of apoptosis, as a measure of early stage cell intoxication, was assessed by examining caspase-3 enzyme activity using the APT165 commercial kit as per manufacturer's instructions (Millipore, Watford, UK). Intestinal tissues were isolated 45 min after exposure to test agents administered by direct intestinal intraluminal injection. Hygromycin (150 μg/mL) was administered as a positive control to incite apoptosis through caspase-3 activation [25].

#### 2.6.1 Microscopy

PIP peptide-mediated uptake of Cy3-labeled insulin (Nanocs) was evaluated in vivo where exposed intestinal segments were isolated 15 min after administration for microscopic analysis. Isolated tissues were rinsed briefly in ice-cold PBS and then fixed with 4% paraformaldehyde on ice prior to assessment using a Zeiss LSM 510 fluorescence microscope. DAPI (4′,6-diamidino-2-phenylindole) was used as a nuclear stain.

#### 2.6.2 Data analysis

Statistical analysis was performed using GraphPad Prism 4.0 software. Data comparisons for dextran transport, Western blot intensity and caspase-3 activity were performed using a two-tailed, un-paired Student's *T*-test. A p-value of < 0.05 was considered significant. Data comparisons for TEER, blood glucose and blood insulin were performed using a one-way ANOVA. Where the ANOVA showed a significant variance between data sets, a Bonferroni post-test was performed to compare PIP peptide data sets to the control data set. A p-value of < 0.05 was considered significant.

## 3 Results

### 3.1 PIP peptide design

#### 3.1.1 Targeting PP1-CPI-17 interactions

Inhibition of MLCP by CPI-17 is driven by PKC-stimulated events with the phosphorylation of residue T^38^ (pT^38^) in CPI-17 enhancing MLCP inhibition over 1000-fold [26]. To target this PP1-CPI-17 interaction, we examined the known structural information (PBD ID: 2RLT) and designed a small cadre of peptides emulating the R^36^VTVKYDRR^44^ sequence in CPI-17 that interacts with PP1 (Table 1). These peptides were synthesized with T^38^ as a focal point due to its phosphorylation potential [15]. Glutamic acid (E) was used to mimic pT^38^; additional basic amino acids were introduced to emulate cell penetrating peptide (CPP) sequences to enhance membrane permeability [27,28] and all d-amino acids in the reverse orientation were used to increase stability [29]. A lead candidate, peptide 640 = rrdykvevrrkkr-NH_2_, was identified.

#### 3.1.2 Targeting PP1-MYPT1 interactions

MLCP inhibition occurs following phosphorylation of residues T^696^ and T^853^ in MYPT1 mediated by Rho-kinase [16,17,30]. MYPT1 binds PP1 via 3 different regions: the RVxF binding motif, the N-terminal arm and the 2nd group of ankyrin repeats of this protein. While phosphorylation of MYPT1 at both T^696^ and T^853^ appears be involved in MLCP regulation, a KVKF sequence within the 300 residue N-terminal domain of MYPT1 facilitates its association with PP1. Analysis of published crystal structures shows that E^300^ to E^309^ of PP1 is positioned between 2 ankyrin repeats of MYPT; in particular the ankyrin repeats bind with Y^305^ and Y^307^, suggesting that the C terminus of PP1 is important for regulatory subunit interaction to mediate isoform specificity. We therefore speculated that a peptide corresponding to the MYPT1 binding motif could prevent MYPT binding to PP1 and hence diminish myosin specificity of the MYPT/PP1 complex. To test this hypothesis we used a strategy similar to that described above for peptide 640 to identify an all d-amino acid peptide referred to as 250: rrfkvktkkrk-NH_2_.

### 3.2 In vitro studies

**Fig. 1:**
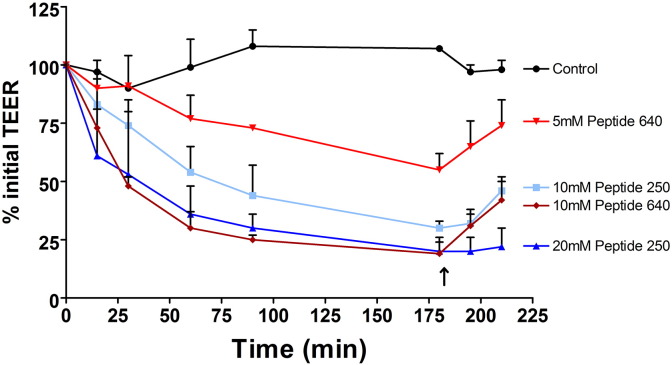
PIP peptides can reversibly affect the trans-epithelial electrical resistance (TEER) properties across confluent, polarized monolayers of Caco-2 cells in vitro. Changes in percentage (%) of initial TEER values measured over time following apical application of PIP peptide 250 or peptide 640 at designated concentrations demonstrated concentration- and time-dependent action. Arrow represents time point when the PIP peptide in the apical compartment was replaced with PBS (washout). Control is where PBS was added to the apical surface with no peptide. Data are means ± SD, n = 6 for control and n = 5 for all peptide groups. One-way ANOVA showed that the data sets were significantly different from each other (p < 0.0001). Bonferroni post-tests showed that 10 mM peptide 250 (p < 0.01), 20 mM peptide 250 (p < 0.001) and 10 mM peptide 640 (p < 0.001) were significantly different from control.

In order to adequately compare PIP 250 and 640, we attempted to match the two peptides based on response changes in TEER [22]. This was done because of several uncertainties that make a direct dose comparison inappropriate since it is likely that the two peptides have different properties critical to their actions: accessibility to their intracellular targets (due to differing CPP capabilities), binding affinities for their respective MLCP-related targets, off-target actions, and intracellular stabilities. We determined that an apical application of 20 mM peptide 250 and 10 mM peptide 640 provided comparable responses as evidenced by changes in TEER (Fig. 1). Reducing the apical dose of these two PIP peptides resulted in dose-dependent changes in TEER with regard to both time of onset and maximum effect. After 180 min of PIP peptide exposure, apical compartment replacement with fresh media initiated reversal of TEER depression that was more rapid for cells exposed to peptide 640 compared to peptide 250. Under all conditions, complete TEER recovery was achieved by 24 h. Similar peptides, with amino acid sequences distinct from PIP peptides 250 and 640, failed to affect Caco-2 monolayer TEER values when tested at 20 mM (data not shown).

**Fig. 2:**
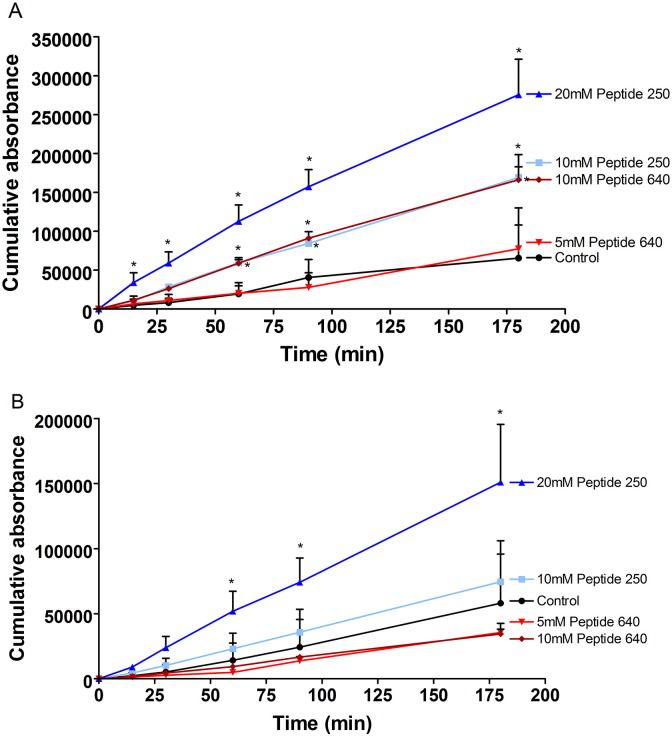
PIP peptide 250 can enhance the uptake of larger solutes than peptide 640 in vitro. Cumulative apical to basal transport of fluorescent dextran across Caco-2 monolayers induced by the apical application of designated concentrations of PIP peptide 250 or 640. (A) 4 kDa dextran transport induced by peptides 250 and 640. (B) 70 kDa dextran transport induced by peptides 250 and 640. PBS added to the apical compartment served as control. Data are means ± SD, n = 6 for control and n = 5 for all peptide treatment groups. * Significantly different from control with two-tailed, un-paired t-test.

We next asked if the actions of PIP peptides 640 and 250 on TEER values translated to changes in paracellular permeability by monitoring the cumulative apical to basal transport of fluorescent dextrans (Fig. 2). Despite producing a nearly 50% loss in TEER after 180 min of exposure, 5 mM of peptide 640 failed to affect 4 kDa dextran permeability. Apical application of 10 mM peptide 640 increased the rate of 4 kDa permeation by ~ 3-fold. Interestingly, 10 mM peptide 640 was equal to 10 mM peptide 250 with regard to 4 kDa flux rates; despite having a slightly delayed and less intense impact on TEER changes. Strikingly, 20 mM peptide 250 resulted in twice the flux rate (~ 6-fold compare to control) for 4 kDa dextran compared to 10 mM peptide 640 even though changes in TEER profiles were nearly identical for these treatments. We also observed that peptide 640 failed to affect the flux of 70 kDa dextran while peptide 250, at 20 mM, could increase this flux ~ 3-fold. The linearity of 4 kDa dextran flux enhancement suggested that permeability changes induced by these PIP peptides was quite rapid despite the time required to achieve a plateau of TEER response (Fig. 1). There was, however, the suggestion of a slightly delayed induction of enhanced 70 kDa dextran transport induced by 20 mM peptide 250. Overall, these results suggest that peptide 640 may induce a more rapid onset but less robust and durable opening of the paracellular route compared to equivalent actions (based upon TEER) induced by peptide 250.

### 3.3 In vivo supression of blood sugar by insulin

**Fig. 3:**
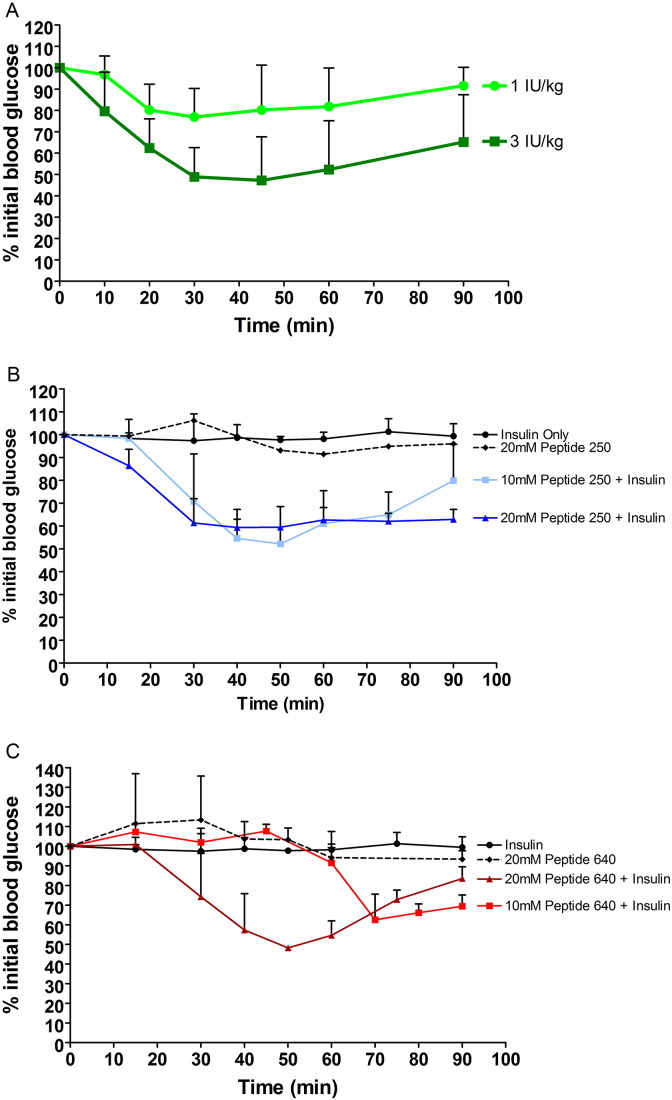
PIP peptides administered with human insulin can suppress serum glucose levels in non-diabetic rats in vivo. (A) SC injection of human insulin resulted in a dose-dependent, reversible suppression of systemic blood glucose levels. Systemic blood glucose levels over time following ILI injection of 30 IU/kg human insulin alone or with (B) peptide 250 or (C) peptide 640. Controls included ILI injection of PIP peptides alone. 10 mM peptide 250 + insulin (p < 0.001), 20 mM peptide 250 + insulin (p < 0.001) and 20 mM peptide 640 + insulin (p < 0.01) were significantly different from insulin only.

Our focus was to examine intestinal epithelial transport prior to addressing formulation challenges related to bypassing stomach acids and pancreatic enzymes; such challenges can be solved with established pill or tablet technologies but require higher order animal models for adequate evaluation. Presently, we used a rat model where a small (50 μL) volume was directly injected into lumen of distal jejunum and proximal ileum segments. It is important to note that the protein–protein interfacial domains being targeted by these PIP peptides are highly conserved between human and rat. We wished to test PIP peptides 250 and 640 at concentrations derived from our in vitro Caco-2 studies and calibrated their actions via intraluminal intestinal injection (ILI) in vivo. Human insulin was selected; it has rapid and easily measured pharmacodynamics (PD) and can be discriminated from endogenous rat insulin by ELISA to derive pharmacokinetic (PK) information. Subcutaneous (SC) insulin injection into non-diabetic rats resulted in a dose-dependent and reversible decline in peripheral blood glucose (Fig. 3A). Insulin injected at 3 IU/kg produced ~ 50% decrease in blood glucose, from 10.1 mM to 4.7 mM, that reached its nadir by ~ 30 min and began to recover by ~ 60 min (Fig. 3A); 1 IU/kg insulin reduced blood glucose from 10.3 mM to 7.9 mM, or ~ 75% of baseline, after 30 min with recovery beginning soon after.

ILI injection of 30 IU/kg insulin had no effect on blood sugar (Fig. 3B), however the same amount of insulin plus 10 mM PIP peptide 250 resulted in a blood glucose drop and recovery profile similar to that observed for the SC injection of 3 IU/kg of insulin (Fig. 3B). PIP peptide 250 dosed at 10 mM with 30 IU/kg insulin reduced blood glucose levels from 10.9 mM to 5.7 mM by 50 min, a drop to 50% of initial blood glucose. PIP peptide 250 dosed at 20 mM with 30 IU/kg insulin reduced blood glucose levels from 14.8 mM to 8.8 mM by 40 min, which was a drop to 60% of initial blood glucose. While recovery to 70–80% of the initial blood sugar level was achieved by 90 min following 10 mM PIP peptide 250 dosing with 30 IU/kg, 20 mM peptide 250 administered with 30 IU/kg by ILI injection resulted in a greater drop in blood glucose by 30 min that remained at this level for the remainder of the 90 min experiment. ILI injection of 30 IU/kg of insulin with 10 mM peptide 640 resulted in a more delayed decrease in blood glucose (Fig. 3C) relative to SC injection (Fig. 3A) or 10 mM peptide 250 (Fig. 3B). ILI injection of 30 IU/kg insulin alone or 20 mM of either peptide 250 or 640 without insulin failed to affect blood sugar levels (Fig. 3B & C).

PIP peptide 640 dosed at 10 mM with 30 IU/kg insulin resulted in reduced blood glucose levels after 60 min, dropping from 10.0 mM to 6.3 mM by 70 min; a decrease to 62.5% of baseline. PIP peptide 640 dosed at 20 mM with 30 IU/kg insulin reduced blood glucose levels after 30 min, dropping from 10.9 mM to 5.2 mM after 50 min; a decrease to 48% of baseline. Blood glucose levels began to return to basal levels by 60 min after ILI injection of peptide 640 tested under these conditions. Thus, ILI injection of 20 mM peptide 640 was required to produce a similar effect and recovery profile as that observed with 10 mM peptide 250 in this in vivo model, but the response appeared to be more dynamic.

### 3.4 Mechansim of PIP peptide actions

These in vivo results are, in general, consistent with our in vitro studies performed using the human intestinal epithelial cell line Caco-2 that suggested a dynamic alteration in paracellular permeability (Figs. 1 & 2). As anticipated, the actions of these PIP peptides in vivo were transient and the duration and time onset of their actions was dose-dependent. Differences between PIP peptides 250 and 640 were readily apparent in vitro where their absolute concentration and duration of exposure could be controlled. We examined the onset and duration of PIP peptides 250 and 640 actions in vivo by determining the phosphorylation status of MLC by comparing the extent of phosphoserine at position 19 of MLC (pMLC) to total MLC in rat intestinal tissue isolated from the sites of PIP peptide exposure over the time course of blood sugar measurements (Fig. 3B & C).

**Fig. 4:**
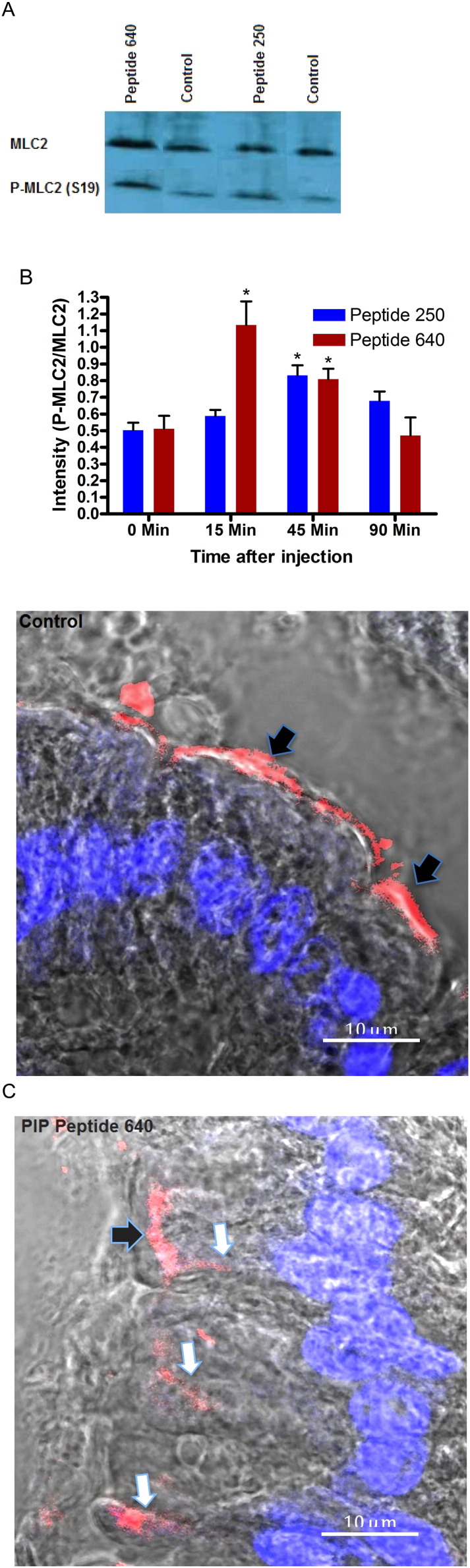
Intraluminal intestinal injection of PIP peptides transiently increases pMLC content and enhances paracellular uptake of insulin in non-diabetic rat intestinal tissue in vivo. (A) Representative semi-quantitative Western blot analysis (at 45 min) used to assess changes in the extent of myosin light chain (MLC) phosphorylated at Ser^19^ (pMLC-Ser^19^) relative to total MLC content. (B) ILI injection of PIP peptides increases pMLC content. Ratios of MLC to pMLC-Ser^19^ levels in rat intestinal tissue at selected time points following apical application of PIP peptides at designated concentrations. * p < 0.05. (C) Paracellular transport of fluorescent (Cy3-labeled) insulin enhanced by PIP peptides following ILI injection. Nuclei are DAPI-stained (blue). Black arrows show label at luminal surface; white arrows show label penetrating between adjacent epithelial cells. Bar = 10 μm.

The ratio of total MLC to pMLC was assessed by semi-quantitative Western blot analysis (Fig. 4A). This analysis demonstrated that the MLC phosphorylation ratio induced by 20 mM peptide 640 was significantly increased by 15 min and remained elevated at 45 min before returning to initial levels at 90 min. The MLC to pMLC ratio profile achieved with 20 mM peptide 250 showed no significant changes at 15 min but was elevated at 45 min before returning to basal levels at 90 min (Fig. 4B). These results correlated well with the time course of blood sugar depression induced by the co-administration of these PIP peptides with insulin (Fig. 3B & C). It is important to note that, while care was taken to isolate MLC from only the intestinal epithelial cells, the extraction procedure used could have resulted in some MLC isolated from other cells present in these isolated intestinal tissues.

We probed our hypothesis of the PIP peptide mechanism of action further by examining the fate of a fluorescent-labeled form of insulin following its ILI injection. Fluorescent (Cy3-labeled) insulin was observed in the intestinal paracellular space only when co-administered with a PIP peptide without gross anatomical modification of the epithelium (Fig. 4C). Together, these results support the hypothesis that apical, topical application of PIP peptides act locally to increase paracellular permeability of rat intestinal epithelium in vivo. Further, the time course of increase paracellular permeability of insulin was consistent with the role of a transient increase of pMLC content in epithelial cells.

### 3.5 Fate of PIP peptide enhanced uptake of insulin

**Fig. 5:**
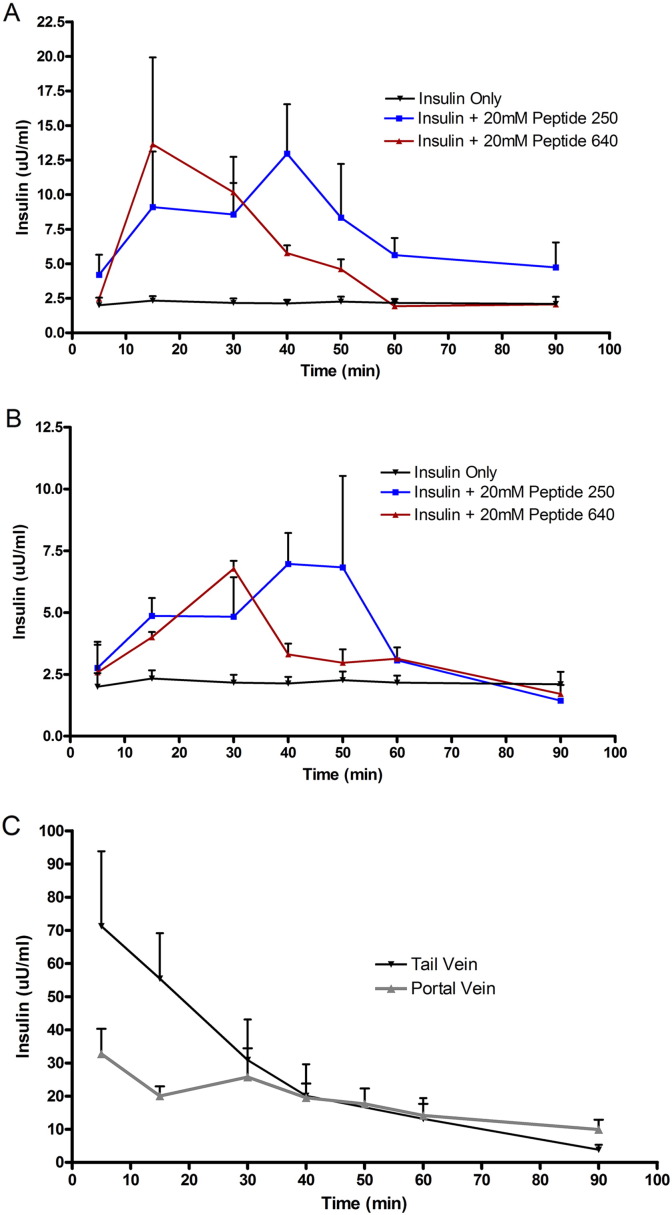
PIP peptides 250 and 640 enhance the uptake of human insulin with different kinetics from the intestinal lumen of non-diabetic rats. (A) Time-concentration profiles of insulin in serum samples from blood collected from the portal vein following ILI injection of PIP peptides with 30 IU/kg of insulin. One-way ANOVA shows data sets are significantly different from each other (p < 0.01). Bonferroni post-tests show that insulin with peptide 250 (p < 0.01) and insulin with peptide 640 (p < 0.05) were significantly different from insulin only. (B) Time-concentration profiles of insulin in serum samples collected from the tail vein following ILI injection of PIP peptides with 30 IU/kg of insulin. There was no significant difference between the groups when compared using one way ANOVA (C) Time-concentration profiles of insulin in in serum samples of blood collected from the tail and portal veins of non-diabetic mice following a SC injection of 3 IU/kg of human insulin. Data are means ± SD for n = 3 for each treatment group.

Time course studies monitoring blood glucose depression and alterations in MLC phosphorylation relevant to total MLC cell content following ILI injection of PIP peptides and  30 IU of insulin suggest an in vivo event window of approximately 30–60 min. To explore this further, we measured serum insulin concentrations in blood collected from the portal vein (Fig. 5A) and the tail vein (Fig. 5B) from 5 to 90 min following ILI injection. Onset of measurable insulin levels and their concentration in the portal vein suggested peptide 640 to incite a more rapid onset of action and shorter duration of action compared to peptide 250, consistent with the time course for phosphorylation ratio changes for these two peptides (Fig. 4).

The total amount of insulin detected by ELISA in the portal and systemic (tail vein) after ILI of 30 IU/kg showed slightly different profiles following enhanced uptake by peptide 250 versus peptide 640 for the conditions tested here. Additionally, we determined time-concentration profiles for portal and systemic concentrations of insulin following SC injection (Fig. 5C). Using non-compartmental analysis, the relative bioavailability of oral administration (relative to SC) for human insulin detected in the portal vein when administered by ILI with peptide 250 versus peptide 640 were 4% and 3%, respectively. Interestingly, the relative bioavailabilies for human insulin reaching the systemic circulation following ILI administration with peptide 250 versus peptide 640 was 1.6% and 1.4%, respectively. These results suggested that a substantial fraction of human insulin delivered to the portal vein did not reach the systemic circulation.

### 3.6 Epithelial cell viability following PIP peptide exposure

**Fig. 6:**
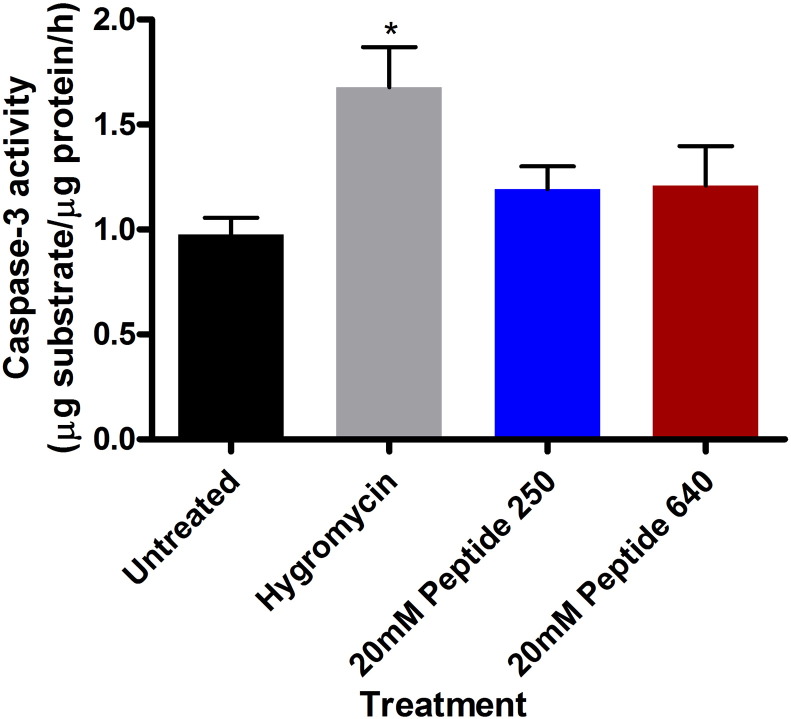
PIP peptides 250 and 640 enhance the uptake of human insulin from the intestinal lumen of non-diabetic rats without induction of apoptosis pathway activation. Caspase-3 enzyme activity in intestinal tissues isolated 45 min after ILI injection of PIP peptides at the designated concentration. Hygromycin (150 μg/mL) was administered by ILI injection to serve as a positive control for the induction of caspase-3. Data are means ± SD for n = 3 for each treatment group * p < 0.05.

Initial in vitro studies using Caco-2 cells suggested that peptide 250 and 640, tested at concentrations that modulated TEER and paracellular permeability, did not affect cell viability as assessed by the mitochondrial membrane polarity marker MTS (data not shown). Due to the transient nature of PIP actions in vivo, we focused on cell signals that might better define early cellular changes that could correlate with decreased cell viability; activation of the caspase enzyme cascade is an early step in apoptosis events in epithelial cells [25]. We measured the level of caspase-3 activity in rat intestinal tissue isolated 45 min after apical exposure of peptides 250 or 640 at concentrations shown to decrease blood glucose (Fig. 3), increase the extent of pMLC (Fig. 4), and enhance the uptake of insulin into the portal vein (Fig. 5). Intestinal tissues failed to show an induction of caspase-3 enzyme activity following similar apical exposure of 20 mM PIP peptide 250 or 640, while hygromycin (150 μg/mL) administered by ILI was used as a positive control for the induction of caspase-3 did result in increasing this enzyme activity (Fig. 6). The concentration of endotoxin in portal vein blood was measured following injection of 20 mM PIP peptide 250 or 640 with insulin or insulin alone. No difference was observed between the treatments (data not shown).

## 4 Discussion

A wide variety of agents have been tested in an effort to increase intestinal paracellular flux for the purposes of enhancing the oral delivery of biopharmaceuticals with quillaja saponin, dipotassium glycyrrhizinate, 18β-glycyrrhetinic, sodium caprate, taurine, and alkylmaltosides being just a few of the agents that have been described [31–33]. In general, these agents were selected empirically through screens involving in vitro cell systems such as Caco-2 monolayers or isolated intestinal tissues [6]. Some of these agents, like palmitoyl carnitine, initially showed promise but ultimately their benefits were correlated with lytic effects on cell membranes that reduced cell viability [32]. Sodium caprate, a medium chain fatty acid present in human milk and approved as an absorption-enhancing agent in a rectal ampicillin suppository causes TJ dilations and enhances paracellular permeability in vitro [34]; the efficacy of caprate in vivo in man, however, is better correlated with non-specific damage to the rectal mucosa rather than paracellular permeability modification [35].

Toxicity, primarily based upon an uncertainty of a specific mechanism of action (or actions), seems to be a central element that limits the identification of agents to safely enhance paracellular transport for the oral delivery of biopharmaceuticals. It appears that transient changes in cellular elements involved in controlling TJ function are not overly toxic. For example, the medium chain fatty acid caprate has been shown to alter expression of the TJ component tricellulin, presumably increasing paracellular flux through disrupted tri-cellular contracts in the epithelium [33]. Further, a novel oily suspension of medium chain fatty acid salts that transiently altered the intracellular distribution of *zonula occludens*-1, a component of functional TJs, to promote absorption by increasing paracellular permeability of the intestinal epithelium has been shown to be safe in monkey toxicity studies [36]. This concept of enhancing paracellular permeability through manipulation of TJ protein function and/or organization correlates well with in vitro and in vivo data showing that a peptide emulating an extracellular loop domain of the TJ protein claudin 1 can similarly enhance paracellular flux [37]. Therefore, methods to selectively and transiently disorganize TJ contacts may provide a potential mechanism of action to effectively increase paracellular permeability.

**Fig. 7:**
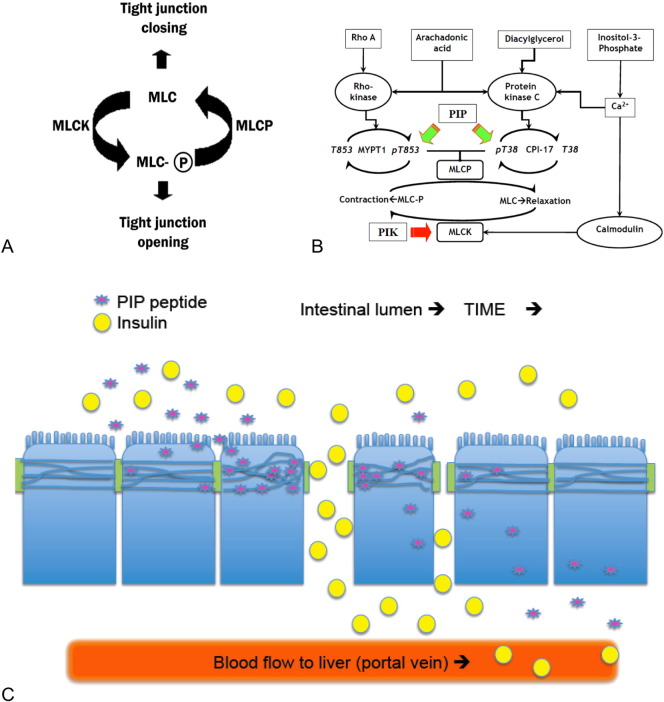
Conceptual aspects of the PIP peptides strategy. (A) General relationship between TJ barrier function and MLC phosphorylation status. (B) Schematic representation of mechanisms regulating the activity of MLC kinase and MLC phosphatase. Ca^2 +^/calmodulin-mediated activation of MLC kinase (MLCK) causes contraction of the actomyosin cytoskeleton associated with TJ structures, resulting in dilation of the space between adjacent epithelial cells–enhanced paracellular permeability. The PIP (permeant inhibitor of phosphatase) peptides (green arrows) described in these studies were designed to disrupt MYPT1 or CPI-17 regulation of MLCP function. PIK (permeant inhibitor of kinase) action on MLCK is noted by a red arrow. (C) Cartoon depicting several of the dynamic factors affecting the PIP peptide-mediated enhancement of insulin uptake. PIP peptide (purple star) entry into epithelial cell and modulation of MLCP function at the TJ to open the paracellular route to solutes, a transient effect due to the systemic uptake, dilution, and elimination. Co-administration of insulin (yellow circles) with a PIP peptide facilitates uptake of this hormone into the portal vein. The location and duration of insulin uptake is dependent upon sufficient adjacent PIP concentration and actions. Movement along the intestinal lumen over time (arrows) and dilution into the luminal contents should affect both PIP peptide actions and the extent of insulin uptake.

We have used two rationally designed agents to incite a defined mechanism of action that leads to the transient opening of intestinal TJs. Our approach is based upon landmark findings made initially by Papenheimer and co-workers that demonstrated the intestinal epithelium to have an endogenous nutrient-activated mechanism for the transient increase in paracellular permeability of solutes [10]. Subsequent studies showed that this increase in paracellular permeation was due to an increase in myosin light chain (MLC) phosphorylation driven by Na^+^-dependent nutrient uptake processes [38]. Validation of MLC phosphorylation in controlling TJ-mediated solute permeability was initially validated using a rationally designed, stable, membrane permeable inhibitor of MLC kinase (PIK) peptide that was shown to be effective in models of chronic epithelial inflammation where unabated MLC kinase (MLCK) activity maintains the epithelia in a hyper-permeable state characterized by increased pMLC levels [12,39]. Presently, we identified similar membrane-permeable, stable, selective inhibitors of MLC phosphatase (MLCP) that counterbalances the actions of residual MLCK activity (Fig. 7A and B). Our rationale was that the topical application of such peptides in vivo would result in a local action on TJ function and that this action would be transient due to dilution and elimination following local action on MLCP activity (Fig. 7C). The cumulative in vitro and in vivo data present in this report is consistent with that concept.

We used information from published crystal structures to design short peptide sequences capable of selectively modulating protein–protein interactions with the goal of emulating interfacial contacts involving MLCP regulatory proteins: CPI-17 and MYPT [16,40]. Investigations over the last 20 years have demonstrated Ca^2 +^/calmodulin to activate MLCK activity while Rho kinase and protein kinase C (PKC) regulate MLCP through MYPT1 and CPI-17, respectively [41]. Additional studies have described the impact of Rho kinase and PKC-zeta on intestinal TJ function [42]. Our design of PIP peptides 250 and 640 addressed several issues associated with using small peptides to target an intracellular protein–protein contact site: peptides that have extensive interfacial contact sites sufficient to achieve target specificity can suffer from poor membrane permeability and are further limited by peptidase-mediated catabolism in the intestinal lumen and epithelial cell cytoplasm [13]. To overcome these issues, we selected protein–protein interfaces that could accommodate peptides prepared from all d-amino acids for increased stability and that would contain an increased positive/negative charge ratio to increase membrane permeability.

Recent studies have validated the approach of using stable, membrane permeable peptides to disrupt an interfacial contact site and alter protein phosphatase 1 in living cells [43]. As previously shown for MLPC modulation with a membrane-permeant peptide designed to target the RVxF-type PP1-binding motif [43] and other cell membrane penetrating peptides [7,44], the PIP peptides examined in our studies did not show significant cytotoxic actions. This is in striking contrast to microcystins, a class of cyclic heptapeptide hepatoxins that inhibit PP1 along with other multiple other Ser/Thr protein phosphatases by binding to a site common to all [45]; illnesses associated with microcystin intoxication are related to non-specific actions resulting from increased phosphorylation of many proteins [46]. While the PIP peptides 640 and 250 are effective and non-toxic, the concentrations required for their function are in the millimolar range with the PIP peptide: insulin ratio being ~ 0.96:0.04. This does not appear to be a strategy-limiting issue since achieving these soluble concentrations of these peptides at local, topical applications where a biopharmaceutical is positioned simultaneously is readily achieved and are in the concentration range currently being employed for empirically derived permeation enhancers. Further, no optimization efforts have yet been performed for these PIP peptides.

We have characterized the extent of insulin that could be transported across the rat intestinal epithelium as a consequence of transiently opening the paracellular route between adjacent epithelial cells. Our studies showed that less than 5% of the material placed in the intestinal lumen could be absorbed. While the peptides used to increase the paracellular permeability were designed to be stable in the enzymatically active environment of the small intestine, the insulin that was co-administered would have been much more labile. Formulation or chemical modification strategies to improve the stability of a co-administered biopharmaceutical should improve this bioavailability outcome during the brief period of action of these PIP peptides. Although increasing the duration and extent of TJ opening using these PIP peptides should also improve this bioavailability outcome, we would be cautious about this strategy from a safety perspective. Interestingly, delivery of a biopharmaceutical, such as insulin, by this route may have added physiological benefit that could compensate for this low bioavailability issue. We observed that approximately half of the absorbed dose present in the portal vein reached the systemic circulation. Such an outcome is consistent with the more physiologically-relevant and previously established insulin-based regulation of glucose levels resulting from pancreatic secretion [47,48].

Overall, our studies have identified a novel strategy to dynamically regulate an endogenous mechanism that controls paracellular permeability in the intestine epithelium and identified two rationally designed PIP peptides: 250 and 640. The 640 peptide was designed to affect a PKC-mediated regulator of MLCP and peptide 250 was designed to affect a Rho-kinase-mediated regulation of MLCP. Interestingly, responses induced by peptide 250 appear to be less dynamic than those affected by peptide 640. The actions of these peptides, however, should be dependent upon a variety of factors that might be different for the two peptides: rate of cell entry, residence time in the cell, and affinity for the intracellular target. Assuming that the potential differences were not overwhelming at the concentrations of PIP peptides used in these studies, our data is consistent with the concept that Rho A activity may mediate slower, more durable TJ changes through its actions on MYPT1 while PKC actions on CPI-17 may provide a mechanism for more rapid and dynamic changes in TJ function for intestinal epithelia function. Further studies, however, will be required to fully test this hypothesis and its potential clinical applications.

## Figures and Tables

**Table 1:** CPI-17 derived peptides tested *.

No.	Peptide sequence	Efficacy
600	NH_2_-RRVTVKYDRR-NH_2_	No
605	NH_2_-RRV**pT**VKYDRR-NH_2_	No
610	NH_2_-RRVTVKY**K**RR-NH_2_	No
615	NH_2_-RRV**pT**VKYKRR-NH_2_	No
620	NH_2_-RR**K**TVKYDRR-NH_2_	No
630	NH_2_-RRV**E**VKYDRR-NH_2_	Yes

*R^36^VTVKYDRR^44^ was selected as the starting sequence (peptide 600). Residues were varied in test series as indicated (bold, underlined). Efficacy was determined by TEER reduction following apical application of 5 mM to Caco-2 cell monolayers with a two-fold increase in 4 kDa dextran flux as the efficacy threshold. Peptide 630 (NH_2_-RRV**E**VKYDRR-NH_2_) was synthesized in the retro-inverso form with d-amino acids as PIP peptide 640 (NH_2_-rrdykvevrr-NH_2_).

## References

[1] Harrison G.A. (1923). Insulin in alcoholic solution by the mouth. Br. Med. J..

[2] Pilkis S.J., Granner D.K. (1992). Molecular physiology of the regulation of hepatic gluconeogenesis and glycolysis. Annu. Rev. Physiol..

[3] Grassi M., Cadelli G. (2001). Theoretical considerations on the in vivo intestinal permeability determination by means of the single pass and recirculating techniques. Int. J. Pharm..

[4] Welling S.H. (2014). The role of citric acid in oral peptide and protein formulations: relationship between calcium chelation and proteolysis inhibition.

[5] Carino G.P., Mathiowitz E. (1999). Oral insulin delivery. Adv. Drug Deliv. Rev..

[6] Deli M.A. (2009). Potential use of tight junction modulators to reversibly open membranous barriers and improve drug delivery. Biochim. Biophys. Acta.

[7] Maher S., Ryan B., Duffy A., Brayden D.J. (2014). Formulation strategies to improve oral peptide delivery. Pharm. Pat. Anal..

[8] Cunningham K.E., Turner J.R. (2012). Myosin light chain kinase: pulling the strings of epithelial tight junction function. Ann. N. Y. Acad. Sci..

[9] Rubas W. (1996). Flux measurements across Caco-2 monolayers may predict transport in human large intestinal tissue. J. Pharm. Sci..

[10] Pappenheimer J.R. (1993). On the coupling of membrane digestion with intestinal absorption of sugars and amino acids. Am. J. Physiol..

[11] Turner J.R. (1997). Physiological regulation of epithelial tight junctions is associated with myosin light-chain phosphorylation. Am. J. Physiol..

[12] Clayburgh D.R. (2005). Epithelial myosin light chain kinase-dependent barrier dysfunction mediates T cell activation-induced diarrhea in vivo. J. Clin. Invest..

[13] Owens S.E., Graham W.V., Siccardi D., Turner J.R., Mrsny R.J. (2005). A strategy to identify stable membrane-permeant peptide inhibitors of myosin light chain kinase. Pharm. Res..

[14] Zolotarevsky Y. (2002). A membrane-permeant peptide that inhibits MLC kinase restores barrier function in in vitro models of intestinal disease. Gastroenterology.

[15] Eto M. (2009). Regulation of cellular protein phosphatase-1 (PP1) by phosphorylation of the CPI-17 family, C-kinase-activated PP1 inhibitors. J. Biol. Chem..

[16] Peti W., Nairn A.C., Page R. (2013). Structural basis for protein phosphatase 1 regulation and specificity. FEBS J..

[17] Terrak M., Kerff F., Langsetmo K., Tao T., Dominguez R. (2004). Structural basis of protein phosphatase 1 regulation. Nature.

[18] Palomo J.M. (2014). Solid-phase peptide synthesis: an overview focused on the preparation of biologically relevant peptides. RSC Adv..

[19] Jbara M., Seenaiah M., Brik A. (2014). Solid phase chemical ligation employing a Rink Amide linker for the synthesis of histone H2B protein. Chem. Commun..

[20] Rubas W., Cromwell M.E., Mrsny R.J., Ingle G., Elias K.A. (1996). An integrated method to determine epithelial transport and bioactivity of oral drug candidates in vitro. Pharm. Res..

[21] Matter K., Balda M.S. (2003). Functional analysis of tight junctions. Methods.

[22] Blume L.F., Denker M., Gieseler F., Kunze T. (2010). Temperature corrected transepithelial electrical resistance (TEER) measurement to quantify rapid changes in paracellular permeability. Pharmazie.

[23] Heim K.E., Morrell J.S., Ronan A.M., Tagliaferro A.R. (2002). Effects of ketamine-xylazine and isoflurane on insulin sensitivity in dehydroepiandrosterone sulfate-treated minipigs (Sus scrofa domestica). Comp. Med..

[24] Saha J.K., Xia J., Grondin J.M., Engle S.K., Jakubowski J.A. (2005). Acute hyperglycemia induced by ketamine/xylazine anesthesia in rats: mechanisms and implications for preclinical models. Exp. Biol. Med. (Maywood).

[25] Pivot X. (2013). Preference for subcutaneous or intravenous administration of trastuzumab in patients with HER2-positive early breast cancer (PrefHer): an open-label randomised study. Lancet Oncol..

[26] Hayashi Y., Senba S., Yazawa M., Brautigan D.L., Eto M. (2001). Defining the structural determinants and a potential mechanism for inhibition of myosin phosphatase by the protein kinase C-potentiated inhibitor protein of 17 kDa. J. Biol. Chem..

[27] Dunican D.J., Doherty P. (2001). Designing cell-permeant phosphopeptides to modulate intracellular signaling pathways. Biopolymers.

[28] Vasconcelos L., Parn K., Langel U. (2013). Therapeutic potential of cell-penetrating peptides. Ther. Deliv..

[29] Fischer P.M. (2003). The design, synthesis and application of stereochemical and directional peptide isomers: a critical review. Curr. Protein Pept. Sci..

[30] Murthy K.S. (2003). Differential signalling by muscarinic receptors in smooth muscle: m2-mediated inactivation of myosin light chain kinase via Gi3, Cdc42/Rac1 and p21-activated kinase 1 pathway, and m3-mediated MLC20 (20 kDa regulatory light chain of myosin II) phosphorylation via Rho-associated kinase/myosin phosphatase targeting subunit 1 and protein kinase C/CPI-17 pathway. Biochem. J..

[31] Cho S.Y. (2002). Enhancement of paracellular transport of heparin disaccharide across Caco-2 cell monolayers. Arch. Pharm. Res..

[32] Duizer E., van der Wulp C., Versantvoort C.H., Groten J.P. (1998). Absorption enhancement, structural changes in tight junctions and cytotoxicity caused by palmitoyl carnitine in Caco-2 and IEC-18 cells. J. Pharmacol. Exp. Ther..

[33] Krug S.M. (2013). Sodium caprate as an enhancer of macromolecule permeation across tricellular tight junctions of intestinal cells. Biomaterials.

[34] Anderberg E.K., Lindmark T., Artursson P. (1993). Sodium caprate elicits dilatations in human intestinal tight junctions and enhances drug absorption by the paracellular route. Pharm. Res..

[35] Lindmark T. (1997). Mechanism of absorption enhancement in humans after rectal administration of ampicillin in suppositories containing sodium caprate. Pharm. Res..

[36] Tuvia S. (2014). A novel suspension formulation enhances intestinal absorption of macromolecules via transient and reversible transport mechanisms. Pharm. Res..

[37] Mrsny R.J. (2008). A key claudin extracellular loop domain is critical for epithelial barrier integrity. Am. J. Pathol..

[38] Turner J.R. (2006). Molecular basis of epithelial barrier regulation: from basic mechanisms to clinical application. Am. J. Pathol..

[39] Mirzapoiazova T. (2011). Non-muscle myosin light chain kinase isoform is a viable molecular target in acute inflammatory lung injury. Am. J. Respir. Cell Mol. Biol..

[40] Sillerud L.O., Larson R.S. (2005). Design and structure of peptide and peptidomimetic antagonists of protein–protein interaction. Curr. Protein Pept. Sci..

[41] Hirano K. (2007). Current topics in the regulatory mechanism underlying the Ca2 + sensitization of the contractile apparatus in vascular smooth muscle. J. Pharmacol. Sci..

[42] Wu L.L. (2011). Epithelial inducible nitric oxide synthase causes bacterial translocation by impairment of enterocytic tight junctions via intracellular signals of Rho-associated kinase and protein kinase C zeta. Crit. Care Med..

[43] McAvoy T., Nairn A.C., Ausubel Frederick M. (2010). Serine/threonine protein phosphatase assays. Current Protocols in Molecular Biology.

[44] Kilk K., Mahlapuu R., Soomets U., Langel U. (2009). Analysis of in vitro toxicity of five cell-penetrating peptides by metabolic profiling. Toxicology.

[45] Campos A., Vasconcelos V. (2010). Molecular mechanisms of microcystin toxicity in animal cells. Int. J. Mol. Sci..

[46] MacKintosh C., Beattie K.A., Klumpp S., Cohen P., Codd G.A. (1990). Cyanobacterial microcystin-LR is a potent and specific inhibitor of protein phosphatases 1 and 2A from both mammals and higher plants. FEBS Lett..

[47] Chap Z. (1987). First-pass hepatic extraction and metabolic effects of insulin and insulin analogues. Am. J. Physiol..

[48] Meier J.J., Veldhuis J.D., Butler P.C. (2005). Pulsatile insulin secretion dictates systemic insulin delivery by regulating hepatic insulin extraction in humans. Diabetes.

